# Characteristics of exonerated cases of child sexual abuse

**DOI:** 10.3389/fpsyg.2026.1794898

**Published:** 2026-03-25

**Authors:** Emily Denne, Colleen E. Sullivan, Tess M. S. Neal

**Affiliations:** 1 Cincinnati Children's Hospital Medical Center, Cincinnati, OH, United States; 2 Gould School of Law, University of Southern California, Los Angeles, CA, United States; 3 Department of Psychology, Iowa State University, Ames, IA, United States

**Keywords:** child sexual abuse, criminal justice, exoneration, false conviction, forensic, recantation, sex offender

## Abstract

**Introduction:**

Exonerations provide insight into how the criminal justice system sometimes fails. Exonerations in child sexual abuse (CSA) cases may be of particular interest as it is often impossible to tell if a crime has truly occurred, many cases lack corroborative evidence, and children’s developmental capacity can at times evoke credibility concerns.

**Methods:**

Using the National Registry of Exonerations, we examined every known exonerated case of CSA in the United States (*N* = 339).

**Results:**

We found these cases were similar to substantiated cases in many ways. Case features included a single victim (67.8%), victim recantation (49.0%), delayed disclosure (47.5%), alleged immediate family member or caregiver perpetration (47.2%), allegations of repeated abuse (47.2%), suggestive questioning (21.8%), custodial dispute issues (19.5%), and satanic ritual abuse allegations (15.0%). Several contained corroborative evidence (28.3%), a notable finding we discuss in detail. We found several case features became less common over time (e.g., satanic ritual abuse/hysteria, suggestive questioning, recantation).

**Discussion:**

While policies guiding the interviewing of children have improved the ways we talk to children, exoneration evidence suggests there is still much to be learned about false accusations and room for improvement. Importantly, case characteristics appear quite similar for true and false allegations. This finding indicates that as interview and investigation protocols have improved in recent years, reducing the likelihood of clear indicators of false allegations, the issues leading to exoneration in CSA cases may now be subtler and more nuanced. As such, case characteristics are not necessarily clear indicators for allegation veracity and every case requires careful attention to detail.

## Characteristics of exonerated cases of child sexual abuse

In March of 2001, a 4-year-old boy described a vivid account of abuse to his mother, claiming he had been sexually assaulted with a pole in his rectum and that parts of his brain had been oozing out of his arms, all while playing in chest-deep snow (University of California Irvine et al., 2022). Samuel Plotnick, a man with whom the child’s mother had been having an affair, was arrested, charged, convicted, and sentenced to 20 years in prison for the crime. The child’s testimony, coupled with physical indicators of assault (substantial bruising), provided clear and compelling evidence that he had been abused. In 2006, new evidence emerged suggesting Plotnick was innocent and that the child’s mother had been pressured by the prosecution to support the child’s testimony. After spending 7 years in prison, Plotnick was exonerated based on misleading forensic evidence, perjury, official misconduct, and an inadequate legal defense (University of California Irvine et al., 2022). While Plotnick’s case was somewhat unique in the fantastical elements of the child’s disclosure, many other child sexual abuse (CSA) exonerations echo these case characteristics – a child with a close relationship with the alleged perpetrator, for example.

Exonerations offer a unique window into understanding how the legal system sometimes fails. An exoneration occurs when a defendant is remitted from blame for the crime. In this study, we examine every known exonerated case of CSA in the United States to gain a better understanding of these wrongful convictions, using archival data from the National Registry of Exonerations. Understanding characteristics in cases of suspected false convictions is an important step in preventing them. Other scholars have examined archival data from the National Registry of Exonerations to better understand some of the general causes of exonerations (see, e.g., Haldeman et al., 2022; Norris et al., 2020; Possley, 2025; Saber et al., 2022), but the current work adds to the literature by focusing narrowly on CSA cases.

### Exonerations and false convictions

False convictions happen and are a troubling byproduct of a criminal justice system, though the U.S. legal system strives to minimize them. The structure of the U.S. legal system sets the stages by which a person progresses from being accused of a crime to becoming a convicted criminal. This process generally involves the identification of a suspect, arrest, charging, pretrial bargaining, and then adjudication, followed by potential appeals processes (Ellsworth and Gross, 2013). This system serves to protect the innocent and punish, incapacitate, and sometimes rehabilitate the guilty. In an effort to limit procedural errors and ensure justice, a defendant has the right to appeal a legal decision once convicted, a process that serves as an important safeguard against wrongful convictions (Hamer, 2014). An appeal occurs when, at the request of the defendant, a lower court’s decision is re-reviewed. The appeals process in the U. S is stringent: appeals are generally limited to procedural errors, and rarely result in exonerations. Instead, when exonerations occur, they typically happen in post-appeal and extraordinary extenuating circumstances, such as the emergence of compelling new evidence (Ellsworth and Gross, 2013).

Appellate decisions usually uphold the original conviction, but occasionally rule that the original decision was erroneous, with the most successful appellate cases leading to a retrial or outright exoneration. However, exonerations are difficult to interpret. They do not guarantee that an actually innocent person was convicted. In some exonerations, the person may have committed the crime, but the evidence was problematic enough or its collection violated technical rules important enough that the court decides to vacate the conviction (LaPorte, 2018). Defendants can be exonerated for numerous reasons including eyewitness misidentification, poor-quality science, false confessions, perjury or false accusations, inadequate legal defense, and misconduct by attorneys, judges, and witnesses (University of California Irvine et al., 2022). In some cases, such as those exonerated through DNA, there is strong evidence that the wrong person has been convicted. In other cases, legal stipulations, such as an inadequate defense, exonerate the accused with less direct evidence of factual innocence.

In the mid-to-late 1980s, DNA testing became available in criminal justice settings (Lynch et al., 2010). The availability of DNA testing allowed the legal system to more conclusively link suspects to the scene of a crime when DNA evidence was found at the scene. However, it also could be used in older post-conviction cases to exonerate actually innocent people who had been serving time for crimes they did not commit (LaPorte, 2018). That is, for some cases in which DNA had been left at a crime scene and preserved as evidence, the science later evolved such that DNA testing was possible to determine whether the convicted person serving time for a crime could be excluded on the basis of their DNA.

DNA evidence serves an important role in exonerations. Post-conviction DNA exonerations challenged basic assumptions about the U. S. justice system, proving that false convictions do indeed happen and on a larger scale than had been realized (Berger, 2006). These false convictions are most often the byproduct of eyewitness misidentification, false confessions, and perjury that were only later discovered as problematic due to DNA exoneration evidence (Gross et al., 2004). The aftermath of the introduction of post-conviction DNA evidence has been an increase in research focused on false convictions (see West and Meterko, 2015). Yet, many have noted great difficulties in studying false convictions, as there is no known ground truth of when and if they have occurred, by which is it impossible to say with certainty if a particular crime has been committed (Lyon et al., 2020). What we do know about false convictions and exonerations generally comes from rape and murder cases, which account for 95% of all exonerations as more DNA is typically found at these types of scenes than other crime scenes (Ellsworth and Gross, 2013).

While the true rate of false convictions will never be definitively known, there have been some estimates. Loeffler et al. (2019) explored self-report measures of innocence in non-capital cases. Prisoner-participants in a state correctional facility in Pennsylvania were asked to anonymously report their criminal involvement. To assess the reliability of the data, collected responses were compared to administrative data. Overall, results revealed an estimated wrongful convictions rate of 6%. We know that exonerations routinely occur, with 158 exonerations in 2024 alone, reaching a total of 3,769 known exonerations today across numerous crimes (University of California Irvine et al., 2025). These exonerations have been spurred in part by the creation of organizations such as the Innocence Project and the Center on Wrongful Conviction, which review cases with evidence suggesting a possible wrongful conviction (Barone, 2017).

### Child sexual abuse exonerations

Exonerations are particularly complicated in CSA cases. Due to the nature of CSA, it is at times impossible to determine with certainty that a crime has occurred. There is often no medical evidence to corroborate a child’s claims and, in many cases, the child may be the only witness (Walsh et al., 2010). Children’s limited memory, suggestibility, and developmental capacity to report specific details of abuse that may be central to the conviction and sentencing of the defendant can further complicate the prosecution of CSA cases (Bala et al., 2001; Goodman and Bottoms, 1993). Credibility concerns are reflected in court, as defense and prosecuting attorneys spend approximately 80% of their questioning assessing and establishing a child’s credibility as a witness (Denne et al., 2019). A wealth of evidence suggests that children can serve as reliable witnesses when questioned appropriately (for a review see Brown and Lamb, 2025). Still, case processing across CSA crimes is generally poorer than case processing across crime in general. That is while many processed cases of CSA result in a conviction, child abuse is less likely to lead to filed charges in the first place (Cross et al., 2003). Of cases that are prosecuted, successful conviction rates are consistent with other violent crimes (Cross et al., 2003). Echoing Cross and colleagues seminal work on prosecution rates, in a more recent examination of 500 cases of reported CSA in a northeastern US state, less than 20% were pursued for prosecution (Block et al., 2023).

While decisions to prosecute are certainly complex, preliminary evidence suggest that decisions not to prosecute may be shaped, at least in part, by perceptions of child credibility. For instance, Cross and Whitcomb (2017) utilized a survey of U.S. prosecutors to examine common reasons for not perusing prosecution in CSA cases. Lack of evidence to corroborate and support a child’s disclosure was the most common reason to decline prosecution. In contrast, cases with suspect confessions (Sumampouw et al., 2020) and cases with more repeated and severe abuse (Walsh et al., 2010) were more likely to be prosecuted. Nevertheless, some successful prosecutions of CSA end in exonerations.

#### False accusation and CSA cases

Through examining known and substantiated cases of CSA, previous research has attempted to establish some risk factors of false accusations. There are some factors, though not certainly diagnostic of malicious false reports or coached lying, that warrant careful, close examination. Historically, child abuse accusations in the midst of custody disputes have elicited concerns about intentional false accusations (Bala and Schuman, 1999; Gardner, 1991). Trocmé and Bala (2005) used a sample of 7,672 child maltreatment investigations in Canada to explore the prevalence of false reports of child abuse. While suspected false reports were more common in custody disputes when compared to the whole sample, they were only suspected to have occurred in 12% of custody cases. Child custody professionals may have particularly strong concerns about parental alienation in these cases, which may influence their case recommendations (Goldfarb et al., 2019). Importantly, children involved in custody disputes may find new opportunities to disclose true accusations of abuse during a custody dispute. Yet their reports may face heightened scrutiny, making them difficult to successfully prosecute (Bala et al., 2001).

In addition to the context of the case, how we talk to children about the abuse they may have experienced is crucial to eliciting accurate reports. Since the infamous McMartin Preschool trials of the 1980s, heightened concerns exist over children’s suggestibility (Bruck and Ceci, 1999; Garven et al., 2000). These trials helped establish that children can, when repeatedly and suggestively questioned, make false allegations of abuse, including CSA (Thompson et al., 1997). The trials also spurred a body of research that attempted to establish the bounds of child suggestibility, showing that children are resilient and less suggestible than previously thought when questioned appropriately (Goodman et al., 1997; Rush et al., 2017). This body of work led to the creation of best-practice guidelines on how to properly question children alleging CSA, emphasizing the importance of open-ended, non-leading questions (Lamb et al., 2007).

Finally, recantations often elicit concerns about child credibility and the accuracy of their reports. Research on recantation can be difficult to interpret due to problems with ground truth, substantiation bias, and suspicion bias which limit much prior research methodologically (Lyon et al., 2020). Yet some methodologically sound research suggests that recantations can happen even in substantiated cases of abuse and thus recantation is not a definitive indicator that the child made an intentionally fabricated or coached report (Malloy et al., 2007). Instead, children may face pressure to recant a claim of CSA, as many reports of CSA involve intrafamilial abuse (Malloy et al., 2007). Recantations are often predicted by poor socio-emotional support for the victim, a longer delay between abuse and disclosure, and when the alleged perpetrator is a parental figure (Baía et al., 2021). Recantations harm perceptions of victim credibility even in true cases of abuse and, as such, warrant careful attention.

### What we can and cannot learn from false conviction data

Researchers examining CSA cases have historically drawn on data from substantiated cases to describe the nature of CSA prosecution and conviction. To complement these studies, we can look to exoneration data to shed light on the characteristics of exonerees. The National Registry of Exonerations provides a record of all known exonerations in the U. S., but there is good reason to believe this dataset is under-representative. The ease with which these cases are found and identified depends, in part, on the publicity they were given (Gross, 2016). It is likely that many more cases remain unidentified. Further, the exonerations captured in the registry tend to be for more severe crimes (e.g., rape, murder, robbery; Gross, 2016), as well as for cases only in the past few decades as the advent of DNA technology made false conviction claims easier to investigate. Thus, this data can provide important information about the reason for exonerations, but it is limited and incomplete. As such, we must be careful when drawing conclusions from this data and do so in light of its limitations. Still, exonerations data clearly show that false convictions occur and are therefore worth studying to glean insight into contributing factors.

## The current study

CSA cases are complicated and require careful and close attention. Exonerated cases can provide some insight into wrongful convictions in allegations of CSA. Researchers to date have generally explored substantiated cases of abuse and neglected to explore cases that have gone on to be exonerated. To address this gap in the literature, we explored and coded the characteristics of every known exonerated case of CSA in the U. S. We did not have *a priori* hypotheses as this research was exploratory and descriptive, and as such the study was not preregistered. We report how we determined data exclusions and how we coded the data, with our materials and coded data available on the Open Science Framework, https://osf.io/3hz2t/?view_only=23d62534c0094471a3a0c1dbc9e6bb5a.

### Methods

Using the National Registry of Exonerations (University of California Irvine et al., 2022) we explored every known exonerated case of CSA as of December 2025. The registry is a comprehensive database of every known exoneration case for all types of crimes in the U. S. (*N* = 3,769), tracked since its establishment in 1989. Limited information is available on how these cases are tracked and entered into the database, but generally the registry collects information about all publicly-known exonerations. The database was created in a collaborative effort between the University of California Irvine, the University of Michigan, and Michigan State University. Each case in the database consists of a page-long summary describing the demographics of the alleged victim and exoneree, characteristics of the alleged crime, and the reason for the exoneration.

We searched the database for any case containing the crime “child sexual abuse.” This search yielded 339 cases, all of which were coded and none excluded. Coders collected information from the case summaries related to the perpetrator/ child relationship, number of victims, mentions of delayed disclosure, mentions of a custodial dispute, details of the specific allegation, whether the child recanted the allegation, mentions of corroborative behavioral or medical evidence, and suggestive interviewing. These case characteristics were based on a combination of (1) what was available in case summaries and (2) what case characteristics were addressed in the published literature on CSA.

We refined and developed these coding categories based on the information available in the summaries of each case. For example, suggestive questioning was coded as such if the case summary explicitly mentioned the use of suggestive questioning from an interviewer or attorney. Recantation was coded as such if the case summary noted that the alleged victim recanted their statement or directly said they were lying about the accusation. Delayed disclosure was coded as such if there was at minimum a week’s delay between when the abuse allegedly occurred and when the child reported it, most often a delay of many years. While there are numerous ways to conceptualize a “delay” in disclosure, we chose 1 week as children who do not report immediately most commonly wait upwards of a year to report (Miller and London, 2020). All coding categories were coded based on whether the case summary explicitly noted that category. Two independent coders coded 187 (55%) of the cases, with high interrater reliability (*K* > 0.90 on all variables). Differences were resolved through discussion. Given the high reliability, one of the coders coded the remaining 152 cases. The coding sheet and our coded data is available on the Open Science Framework.^1^ Below we report descriptive results collectively, as well as stratified by year, child age, reason for exoneration, and corroborative evidence type. We did not report inferential statistics, as group sizes across stratified groups were too small to meaningfully analyze and interpret.

## Results

**Figure 1:**
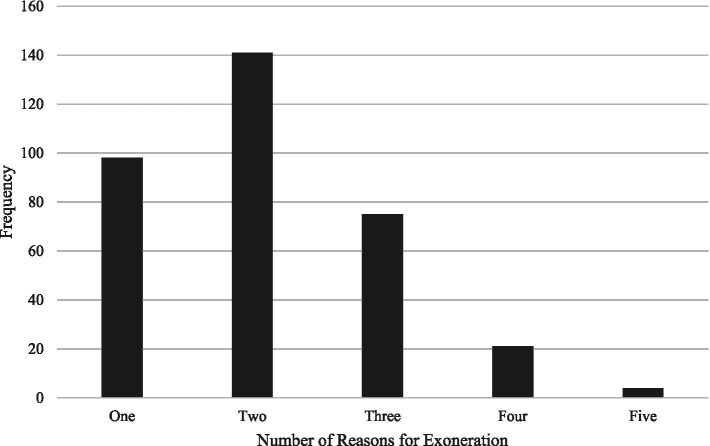
Number of reasons for exoneration per child sexual abuse case. Across 35 of these cases, DNA evidence contributed to an exoneration.

Many of these cases were concentrated in Texas (*n* = 50), California (*n* = 40), Michigan (*n* = 23), Washington (*n* = 21), New York (*n* = 19), Ohio (*n* = 17), Wisconsin (*n* = 13), and North Carolina (*n* = 12; all other states had less than 10 exonerations). For each case, there were between one and five reasons for exoneration (see Figure 1), including perjury or false accusation, official misconduct, inadequate legal defense, false or misleading forensic evidence, false confession, and mistaken witness identification. Children at the age of discovery of abuse (often through disclosure from the child or a witness) ranged in age from 21 months to 18 years (*M* = 9.06, *SD* = 3.62; 45 cases were excluded for unclear details on ages of children included in the case, often because there were multiple children involved), and alleged perpetrators at the time of the accusation ranged in age from 13 to 69 (*M* = 32.90, *SD* = 11.00; for one exoneree their age was unclear). The majority of alleged perpetrators were White (59.0%; 27.4% African American, 11.2% Hispanic, 1.2% Asian, 0.6% other) and predominantly male (87.9%; 12.1% female). Among female exonerees, one-fifth involved satanic ritual or daycare related cases in which numerous exonerations occurred. All convictions occurred between 1974 and 2023 (*M* = 1997; *SD* = 10.28) and all exonerations occurred between 1989 and 2025 (*M* = 2008; *SD* = 9.62) (Table 1).

**Table 1:** Frequency of reasons for exoneration with definitions.

Reason for exoneration	Definition	*N*	%
Perjury or false accusation	*“A person other than the exoneree committed perjury by making a false statement under oath that incriminated the exoneree in the crime for which the exoneree was later exonerated, or made a similar unsworn statement that would have been perjury if made under oath.”*	288	85.0%
Official misconduct	*“Police, prosecutors, or other government officials significantly abused their authority or the judicial process in a manner that contributed to the exoneree’s conviction.”*	148	43.7%
Inadequate legal defense	*“The exoneree’s lawyer at trial provided obviously and grossly inadequate representation.”*	106	31.3%
False or misleading forensic evidence	*“Faulty or misleading expert or forensic evidence may have led to a factually erroneous conclusion, at any stage of the investigation or adjudication, that contributed to the defendant’s false conviction.”*	98	28.9%
Mistaken witness identification	*“At least one witness mistakenly identified the exoneree as a person the witness saw commit the crime.”*	40	11.8%
False confession	*“A confession is a statement made to law enforcement at any point during the proceedings which was interpreted or presented by law enforcement as an admission of participation in or presence at the crime, even if the statement was not presented at trial. A statement is not a confession if it was made to someone other than law enforcement. A statement that is not at odds with the defense is not a confession. A guilty plea is not a confession.”*	29	8.6%

Definitions directly quoted from the National Registry of Exonerations (University of California Irvine et al., 2022).

**Figure 2:**
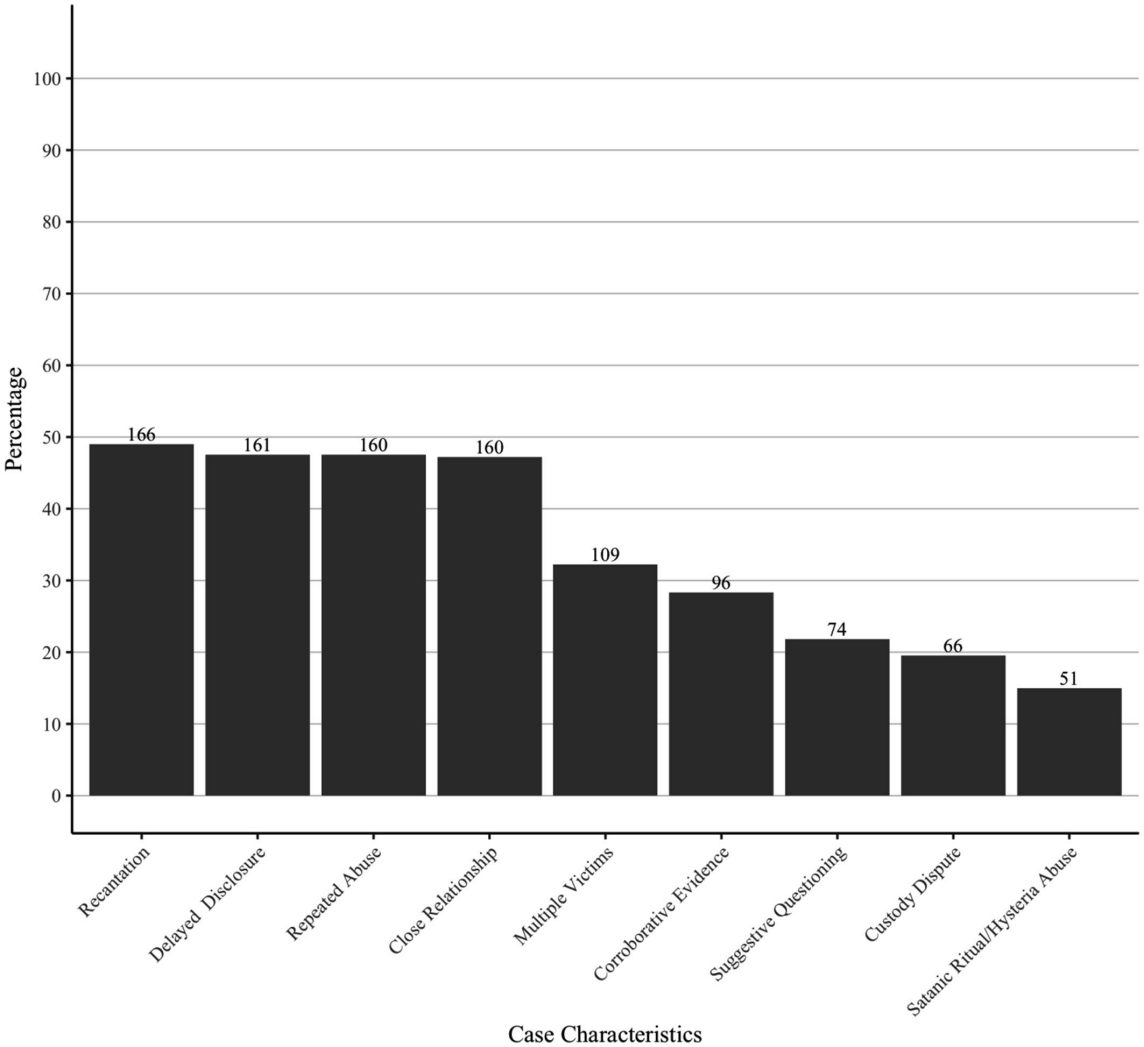
Frequency of various characteristics in exonerated cases of child sexual abuse. Frequency of case characteristics in all known child sexual abuse (CSA) convictions that later resulted in exoneration in the United States (339 exonerated cases of CSA in the National Registry of Exonerations as of December 2025).

See Figure 2 for a visualization of the coded data regarding the frequency of various characteristics of exonerated CSA cases. Over half of these cases included a single victim (67.3%), victim recantation (49.0%), allegations of repeated abuse (47.5%), and delayed disclosure (47.5%). Suggestive questioning was present in 21.8% of cases. A number of cases occurred in the context of a custody dispute (19.5%) or involved satanic ritual abuse allegations (15.0%). The alleged perpetrator in the majority of cases was an immediate family member or caregiver (47.2%; *n* = 160). Other alleged perpetrators were a family friend or well-known other (15.3%; *n* = 52), day care worker or non-caregiver employee in the family home (12.7%; *n* = 43), extended family member (9.1%; *n* = 31), stranger (14.2%; *n* = 49), or was unclear (1.5%; *n* = 5) (Table 2).

**Table 2:** Reason for exoneration in CSA cases by corroborative evidence type.

Reason for exoneration	Behavioral *n =* 16	Medical		
		STI *n =* 11	Physical trauma *n =* 48	Other *n =* 36
Perjury or false accusation	12 (75.0%)	9 (81.8%)	40 (83.3%)	31 (86.1%)
Official misconduct	3 (53.0%)	8 (72.7%)	22 (45.8%)	20 (55.6%)
Inadequate legal defense	6 (37.5%)	5 (45.5%)	23 (47.9%)	16 (44.4%)
False or misleading forensic evidence	7 (43.8%)	2 (18.2%)	17 (35.4%)	7 (19.4%)
Mistaken witness identification	5 (31.3%)	0 (0.00%)	9 (18.8%)	6 (16.7%)
False confession	1 (6.3%)	4 (36.4%)	5 (10.4%)	1 (2.8%)

In 28.3% of cases, corroborative evidence was present, such as medical or behavioral evidence testified to by a professional (e.g., sexually transmitted infection, physical evidence of trauma). Of the 96 cases that contained corroborative evidence, 91 (94.8%) contained medical evidence and 16 (16.7%) contained behavioral evidence (some cases contained both). Of cases that contained medical evidence, 48 (52.7%) contained evidence of physical trauma, 11 (12.1%) involved a sexually transmitted infection, and 36 (39.6%) contained some other form of medical evidence (e.g., doctor indicated unspecified evidence of abuse). Of the 11 cases with evidence of a sexually transmitted infection, the child often identified another perpetrator later on. Cases containing behavioral evidence or medical evidence most often resulted in an exoneration through perjury or false accusation. A large proportion of cases with medical evidence also resulted in an exoneration due to official misconduct (Table 3).

**Table 3:** Characteristics of exonerated CSA cases by child age.

Case characteristics by age group	0–3 years *n =* 20	4–7 years *n =* 112	8–11 years *n =* 135	12–15 years *n =* 76	15–18 years *n =* 28
Repeated abuse	12 (60.0%)	60 (53.6%)	76 (56.3%)	28 (39.8%)	10 (35.7%)
Recantation	6 (30.0%)	66 (58.9%)	83 (61.5%)	26 (34.2%)	10 (35.7%)
Delayed disclosure	11 (55.0%)	47 (42.0%)	70 (51.9%)	30 (39.5%)	14 (50.0%)
Multiple victims	8 (40.0%)	56 (50.0%)	58 (43.0%)	22 (28.9%)	3 (10.7%)
Corroborative evidence	14 (70.0%)	50 (44.6%)	23 (17.0%)	17 (22.4%)	4 (14.3%)
Suggestive questioning	6 (30.0%)	28 (45.0%)	26 (19.3%)	1 (12.2%)	2 (7.2%)
Satanic ritual/hysteria abuse	1 (5.0%)	19 (17.0%)	13 (9.6%)	1 (1.3%)	0 (0.0%)
Custody dispute	4 (20.0%)	26 (23.2%)	42 (31.1%)	11 (14.5%)	0 (0.0%)

Cases that contained children with unclear ages, i.e., where the child’s age was specified generically as “child” or “teenager” were excluded from analyses, *n* = 45.

To explore possible pattens across case characteristics that may be related to the age of the alleged victim, we stratified these cases by alleged victim age (0–3 years, 4–7 years, 8–11 years, 12–15 years, 15–18 years; Table 4). Age groups were determined based on Eg et al. (2024) work examining age differences in the prosecution of CSA cases. The youngest children (age 0–3) had the highest proportion of cases with corroborative evidence, repeated abuse, and delayed disclosure. Children in the middle age brackets (ages 4–7 and 8–11) accounted for almost all of the satanic ritual abuse/ hysteria cases and most recantations. Children in the oldest age brackets (ages 12–15 and 15–18) had the smallest proportion of repeated abuse, satanic ritual abuse, suggestive questioning, and custody dispute related cases.

**Table 4:** Characteristics of exonerated CSA cases in ten-year intervals by year of conviction.

Case characteristics	1970–1980 *n* = 4	1981–1990 *n* = 89	1991–2000 *n* = 123	2001–2010 *n* = 74	2011–2020 *n* = 47	2021–present *n* = 2	Overall *N* = 339
Repeated abuse	3 (75.0%)	42 (47.2%)	61 (49.6%)	35 (47.3%)	18 (38.3%)	1 (50.0%)	160 (47.2%)
Recantation	0 (0.0%)	44 (49.4%)	76 (61.8%)	30 (40.5%)	15 (31.9%)	1 (50.0%)	166 (49.0%)
Delayed disclosure	0 (0.0%)	33 (37.1%)	63 (51.2%)	39 (52.7%)	26 (55.3%)	0 (0.0%)	161 (47.5%)
Multiple victims	1 (25.0%)	46 (51.7%)	40 (32.5%)	17 (23.0%)	5 (10.6%)	1 (50.0%)	109 (32.2%)
Corroborative Evidence	1 (25.0%)	31 (34.8%)	42 (34.2%)	17 (23.0%)	3 (6.4%)	1 (50.0%)	96 (28.3%)
Suggestive questioning	0 (0.0%)	38 (42.7%)	21 (17.1%)	9 (12.2%)	5 (10.6%)	1 (50.0%)	74 (21.8%)
Satanic ritual/hysteria abuse	0 (0.0%)	29 (32.6%)	21 (17.1%)	1 (1.4%)	0 (0.0%)	0 (0.0%)	51 (15.0%)
Custody dispute	0 (0.0%)	11 (12.4%)	30 (24.4%)	19 (25.7%)	6 (12.8%)	0 (0.0%)	66 (19.5%)

To examine various patterns of case characteristics and understand how these patterns may have changed over time, we broke down the cases by year of conviction in 10-year intervals (1970–1980, 1981–1990, 1991–2000, 2001–2010 and 2011–2020, and 2021- present). The data suggest that several of the more problematic features of cases that might lead to false convictions became less common over time (e.g., satanic ritual abuse/hysteria cases, suggestive questioning, recantation).

Most cases (*n* = 241, 71.1%) contained more than one reason for exoneration. Perjury or false accusation was the most common reason for exoneration and was documented in 288 cases (85.0%). Yet many of these cases contained a second, third, or fourth reason for exoneration (*n* = 205, 60.5%). Still, a subset of defendants were exonerated due to perjury or false accusation (*n* = 82, 24.2%) alone, which we explored further.

### Perjury or false accusation cases as a sole reason for exoneration

There were 82 cases that were exonerated due to perjury or false accusation alone. Perjury occurred when a person other than the exoneree made a false statement under oath that incriminated the exoneree. We explored these cases as they are inherently less complex than cases with multiple reasons for exonerations. Simultaneously, these cases are more likely to represent factual innocence based on reason for exoneration alone (whereas official misconduct, for example, might not always represent factual innocence). These perjury or false accusation cases were included in all prior analyses but are being examined on their own here, in an effort toward better understanding one reason for exoneration that occurs with some regularity.

Many of these cases (46.3%; *n* = 38) involved allegations of repeated abuse, 34.2% (*n* = 28) contained allegations of only a single instance of abuse, and the remainder (20.7%; *n* = 17) were unclear. Many of the cases (42.7%; *n* = 35) involved recantation. Clear evidence of delayed disclosure occurred in 46.3% of these cases (*n* = 38). Most involved a single alleged victim (70.7%; *n* = 58) and the remainder involved multiple alleged victims (30.5%; *n* = 25). Some cases (17.1%; *n* = 14) involved corroborative evidence. Suggestive or repetitive questioning was explicitly mentioned in 14 cases. Satanic ritual abuse or hysteria abuse occurred in 12. Several of these exonerated cases occurred specifically in custody dispute or parental dispute cases (20.7%, *n* = 17). These patterns are similar in many ways to characteristics of cases across the complete sample, and to the typical characteristics of CSA cases with standing convictions.

## Discussion

This is the first study, as far as we are aware, that examines every known, exonerated case of CSA in the U. S. to better understand these cases and what characteristics are present in cases of alleged sexual abuse of children. It is highly probable that some of these exonerations are cases of true innocence, whereas others are not. We cannot definitively know which cases are true innocence exonerations and which are not. Yet, this body of information is worth closely examining.

Many of these cases reflected qualities of CSA cases in general. In most cases of CSA, the perpetrator is a known adult in patterns that are similar in some ways to those that emerged in this set of exonerated cases. For instance, Denne et al. (2019) explored 134 cases of 5 to17-year-olds alleging sexual abuse in Arizona courts, finding the defendant was a close adult (e.g., family member, friend, babysitter) in 95% of cases. Similarity, in our exonerations data, only about 15% of alleged perpetrators were strangers. Many exonerated cases (48.1%) involved allegations of repeated abuse. In an examination of CSA cases through the reports of 5- to 17-year-olds in Scottish courts, 71% of children made allegations of repeated abuse – a similar but higher proportion than that of these exonerated cases (Szojka et al., 2017). And like in cases of abuse where the ages of victims vary, the ages of the children in this set of exonerated cases varied. Additionally, like many convicted cases of CSA, many exoneration cases involved delayed disclosure (Hershkowitz et al., 2007). Our findings indicate that these elements are not clear indicators of lying or truth, but could be consistent with either. Still, we must be cautious when comparing exonerated to prosecuted CSA cases, as the latter could contain future exonerations.

The context of these allegations can provide some further insight. Custody or parental dispute-related issues appeared in nearly one fifth of wrongful convictions (19%), a slightly higher proportion than the 12% found by Trocmé and Bala in their 2005 study of false reports of child abuse in thousands of cases (although their findings included numerous forms of abuse, of which children were least likely to make an intentionally false claim of sexual abuse). No clear pattern emerged demonstrating how this may have changed over the years. Again, it is important to note that these cases may face increased scrutiny due to the complexities of navigating CSA allegations in the midst of a custodial dispute. In isolation, an allegation in the midst of a custody dispute tells us little about the credibility of a child’s report. Instead, examining these cases within the larger framework within which they occur can inform assessment of child credibility. It is also worth considering that children may find new opportunities to disclose true abuse in the context of a custody dispute (for instance, when an offending caregiver leaves the home or is about to reenter the home). Indeed, evidence suggests that disclosure of abuse is notably difficult when the offender lives with the victim—a barrier to disclosure (Leclerc and Wortley, 2015).

Between 1981–1990 and 1991–2000, the bulk of CSA exonerations occurred. These year brackets contained almost all of the satanic ritual abuse cases as well as a large proportion of the cases containing suggestive questioning. Most satanic ritual abuse cases also contained suggestive questioning as the two are generally conflated. The hysteria resulting from widespread media coverage on satanic ritual abuse cases spurred a great deal of research on memory, ritual abuse, and questioning practices (Bottoms and Davis, 1997) that likely contributed to the high number of exonerations of these cases between 1981–2000.

As noted, satanic ritual abuse and daycare hysteria abuse appeared in about 15% of cases, occurring in the late 1980s and early 1990s. These cases almost exclusively contained children in the 4–11 year age range. These cases were somewhat distinct from the others in that they frequently involved other characteristics associated with false allegations: improbable allegations (e.g., being thrown in the ocean to sharks) and highly suggestive questioning techniques. Of course, true allegations of abuse can, at times, contain fantastical or impossible details. For instance, children may incorporate threats that the perpetrator has made into their narrative when recounting abuse that has truly occurred (Everson, 1997). Improbable and fantastical elements in a child’s disclosure should not necessarily discredit their whole report, but instead suggest the need for further questioning and investigation. Still, fantastical and false details can be the result of suggestive questioning.

In light of many of these cases, our understanding of satanic ritual and daycare abuse has advanced substantially; these cases are unique and distinct from more typical allegations of CSA. Given the current political climate in the U. S. with the prevalence of conspiracy thinking in the public (Walter and Drochon, 2020), including some people believing that a Satanic pedophile ring is active in certain political and societal circles (Vrzal, 2020), it is possible that CSA allegations might arise that are similar to those that arose in the infamous McMartin Preschool Trials of the 1980s (see, e.g., Balko, 2025). Armed with better knowledge of children’s suggestibility and better methods for questioning potential victims of abuse, we might hope to better protect against false convictions in cases today.

Interestingly, about a third of the exonerated cases contained corroborative medical or behavioral evidence, and half of the cases with corroborative evidence also contained a recantation (*n* = 48, 48.5%). Further, 70% of cases containing the youngest alleged victims (age 0–3 years) also contained corroborative evidence. We examined cases with corroborative evidence specifically, as these cases provide compelling evidence that a crime was committed, whereas cases without corroborative evidence generally rely solely on a victims report. CSA cases do not always contain medical evidence (Kreston, 2007), yet many of the cases in the exoneree database had corroborative evidence – a trend which persisted across the years. This may be a function of our sample, as cases with corroborative evidence may be more likely to reach court generally. When corroborative evidence is presented, it is not always clear how it should be interpreted. Corroborative evidence in these cases included both behavioral evidence (e.g., Child Sexual Abuse Accommodation Syndrome, Post-Traumatic Stress Disorder) and medical examination evidence (e.g., damages to hymen, anal scarring). Eleven cases specifically involved the presence of a sexually transmitted infection in at least one victim. Of note, in many of these cases the child later on identified another perpetrator or compelling evidence (e.g., DNA) emerged which exculpated the accused. Some degree of controversy exists over the reliability of different forms of medical evidence available in CSA cases. Research suggests that medical findings in CSA cases are not always objective, reliable, or based in recent research (Pillai, 2007). Of all cases containing corroborative evidence, most were exonerated due to perjury or false accusation (*n* = 92) or official misconduct (*n* = 53). Perjury cases provide more compelling evidence that a crime occurred and the wrong suspect imprisoned, while official misconduct provides less direct evidence for true acquittal. Here, interpreting exonerations that contain corroborative evidence and official misconduct alone, should be done so cautiously.

The Bernard Baran illustrates the complexity of corroborative evidence. In 1984 Baran, an employee at an early childhood developmental center, was charged with sexually assaulting several children at the center after one child tested positive for gonorrhea of the throat. Evidence later emerged documenting highly suggestive and coercive questioning from interviewers leading to Baran’s exoneration. Still, the presence of a sexually transmitted infection suggests another perpetrator. Thus, we learned from some of these cases that while corroborative evidence may be consistent with abuse having occurred, it is not always useful for implicating the particular suspect in question.

Behavioral evidence is a powerful predictor of conviction in CSA cases, despite medical and behavioral evidence in CSA cases being complicated and difficult to interpret (Adams et al., 1994; Lewis et al., 2014). Evidence suggests that many cases of CSA are judged by mental health professionals to be true when there is behavioral evidence to corroborate abuse, even in the absence of a child’s disclosure (Herman, 2010). Additionally, while some of the medical evidence in these cases were arguably indisputable indicators that abuse occurred, such as the presence of a sexually transmitted infection in a young child, other pieces of medical evidence, like vaginal scarring, can be difficult to interpret and do not prove that the specific events alleged are what actually occurred (Frasier and Makoroff, 2006). Particularly with older cases in our sample, some forms of medical evidence have been discredited as indicators of abuse across the years (e.g., an anus wink response such as in the case of Brenda Kniffen). Researchers would do well to further establish the bounds of credibility and reliability for medical and behavioral evidence presented in CSA cases. These findings have potential implications for policy which could reflect the inconsistency and complexity of medical evidence in these cases. Particularly as no pattern emerged across the years, we may still have room for improvement when evaluating medical evidence in CSA cases.

About half of these cases contained a partial or full recantation of the child’s testimony, and recantations were most common among children in the middle age groups, ages 4–11 years old. Of note, 79.5% of recantations in this study were not satanic ritual abuse cases. Still, as we see from corroborative evidence in many of these cases, it is possible there was a different perpetrator or the perpetrator was wrongfully acquitted. Importantly, 82.4% of these recantations occurred post-conviction, often when the child was an adult, suggesting a recantation pretrial or during the trial, when a child is facing pressure to recant, may represent something different than a recantation years later. Our findings, coupled with prior evidence (Malloy et al., 2007), suggests that recantation during trial may be more likely to reflect familial or other pressures to recant, while recantation as an adult may possibly reflect a true recantation. Policy could accommodate the different motivators for recantation, and work in ways to prevent recantation in true abuse cases and hold space for them in true denials. Still, due to the limitations of this dataset, we cannot draw strong conclusions about recantation. More systematic work can be done to better explore recantation in these sensitive cases, such as work by Malloy et al. (2007) suggesting that recantations are not necessarily indicative of a false disclosure.

Finally, many but not all of these cases involved suggestive questioning practices. Suggestive questioning practices were proportionally most common across children ages 4–7, whose communication skills are still developing. Our understanding of children’s suggestibility has advanced substantially since the 1980s and 1990s (Ceci and Bruck, 1993). We observed that suggestive questioning was much more prevalent in earlier cases than in more recent cases. We know that suggestive questioning can lead to false allegations (Ceci and Bruck, 2006), as we see in many of these cases. Interestingly, suggestive questioning frequently resulted in claims that were somewhat unbelievable. This was particularly true in the satanic ritual cases. For example, in one case that resulted in acquittal, children made allegations that the defendant had violently killed animals in their presence and buried children near a playground when the children were attending daycare (University of California Irvine et al., 2022). Importantly, highly suggestive questioning was frequently mentioned in the acquittal, suggesting it undermines a child’s credibility in cases where abuse has truly occurred as well.

### Limitations

There are numerous limitations to this sample which are important to mention and place our results in context. Some of these cases are relatively old, dating back over 30 years. There have been substantial changes in our understanding of children as victims and witnesses since the 1990s, including advances in policy related to the forensic questioning of children. Child advocacy centers and protocol on interviewing children have greatly improved the ways in which we talk to children about the abuse they have experienced and have likely impacted the rates of false reports (Newman et al., 2005). Indeed, many of these early cases and exonerations spurred a body of research on understanding children’s capacities in court. Yet some of these false convictions were as recent as 2023, suggesting there is still much to be learned about false accusations and room for policy improvement.

Additionally, we have no way of definitively knowing if exonerations reflect actual innocence. There are various ways that a defendant can end up in the database as exonerated. Particularly for those exonerated through official misconduct or inadequate legal defense, it is possible that the defendant was exonerated even if they had committed the crime and were not actually innocent. There may also be wrongfully convicted defendants whose cases have not been successfully appealed. This precludes us from making any strong inferences about our data. In addition, this sample is not exhaustive – it does not include many other cases where the CSA charged was successfully appealed. Further, a comparison group of true cases of abuse would allow us to draw more concrete conclusions from this dataset. Unfortunately, we could locate no publicly available datasets for comparative purposes.

Further, it is possible that exonerated cases tend to reflect characteristics of cases that are more likely to be accepted for prosecution generally. These included cases with medical evidence and cases involving repeated abuse. In addition, cases with medical evidence may have provided an avenue for appeal which cases without medical evidence lacked. Finally, a core challenge of examining these exonerations lies in the limited information available for each case. While each case contains a written case summary, some of those summaries were more detailed than others and we only had access to the information provided in these summaries. More complex case details such as aspects of the allegation that changed over time or greater detail about the characteristics of both the alleged victim and accused are sometimes missing from the case summaries. Additionally, many of the factors we coded for were not reported systematically across cases, but rather informally across case summaries. As a result, our findings may be under-inclusive and we may be missing informative details not included in the case summaries. We are only able to make cautious inferences based on a limited summary of the cases.

## Conclusion

Understanding characteristics of false allegations is an important step to better ensure true cases are successfully prosecuted and false cases are acquitted. Critically, these exoneration cases underscore issues with the criminal justice system and highlight ways in which the system has failed defendants and, in some cases, victims. Specific cases, as well as the database collectively, draw attention to these flaws in the system. To highlight a specific case, Jepheth Barnes was accused in 1995 of sexually abusing an 11-year-old girl. Sperm was uncovered by the emergency room physician who examined the girl. While there was insufficient evidence to obtain a full DNA profile, Barnes could not be ruled out. He was convicted and sentenced to 16 years in prison. DNA evidence later exonerated Barnes and instead incriminated the child’s older brother. This case is a clear example of how the criminal justice system failed both the defendant and victim where evidence indicated a crime had occurred, but the charged defendant was not the perpetrator. While it is often unclear whether a crime has truly occurred in CSA cases, particularly when the defendant is exonerated, the narratives of wrongfully convicted defendants and the accusing victims highlight how complicated these cases are, with consequences for all.

To date, researchers exploring the characteristics of CSA claims have primarily looked at substantiated cases and largely overlooked exonerations. Our paper begins to fill this gap, finding that these cases, in many ways, are similar. While CSA interviewing and investigating practices have improved due to a wealth of research (for example the NICHD protocol, Lamb et al., 2007), our findings suggest that the issues leading to exoneration may be subtle. We found that many recent exoneration cases did not have clear indicators of false allegations. That is, characteristics of cases with true and false allegations were overlapping in many ways.

To avoid convicting wrongfully accused defendants and subsequent exonerations, policing agencies and legal practitioners should perform quality interviews with children and follow investigation best-practices. The presence of certain case characteristics alone (e.g., recantation, a custody dispute, known perpetrator accused) are not clear indicators that an allegation is either true or false. Instead, each case - regardless of particular case characteristics - deserves careful investigation, and the totality of evidence towards innocence and guilt must be considered.

We urge researchers to continue refining and advancing best practice when interviewing children, particularly in cases that face heightened scrutiny due to a recantation or custodial dispute. This is important to ensure innocent defendants are not convicted, but also, that guilty defendants are not exonerated due to complex family dynamics, false recantations, and bad science practices that make a child’s claims less credible. Creating a clearer picture of false accusations is critical in ensuring safety and justice for both children and the people accused of sexually assaulting them. In addition, researchers should continue to examine how community pressures to convict the accused influences case outcomes, and examine each stage of the investigation and trial process to assess the different points at which mistakes can be made and miscarriages of justice can be perpetuated.

## Data Availability

The original contributions presented in the study are included in the article/supplementary material, further inquiries can be directed to the corresponding author/s.

## Footnotes

1
https://osf.io/3hz2t/?view_only=23d62534c0094471a3a0c1dbc9e6bb5a
